# Supplementary material for: Unexpected Inhibitory Effect of Octenidine Dihydrochloride on Candida albicans Filamentation by Impairing Ergosterol Biosynthesis and Disrupting Cell Membrane Integrity
Source: Antibiotics (Basel). 2023 Nov 28;12(12):1675. doi: 10.3390/antibiotics12121675 (PMC10741164; doi:10.3390/antibiotics12121675)
Supplement: Supplementary file 1 [file antibiotics-2708451-supplementary.pdf › antibiotics-2708451-supplementary.pdf]

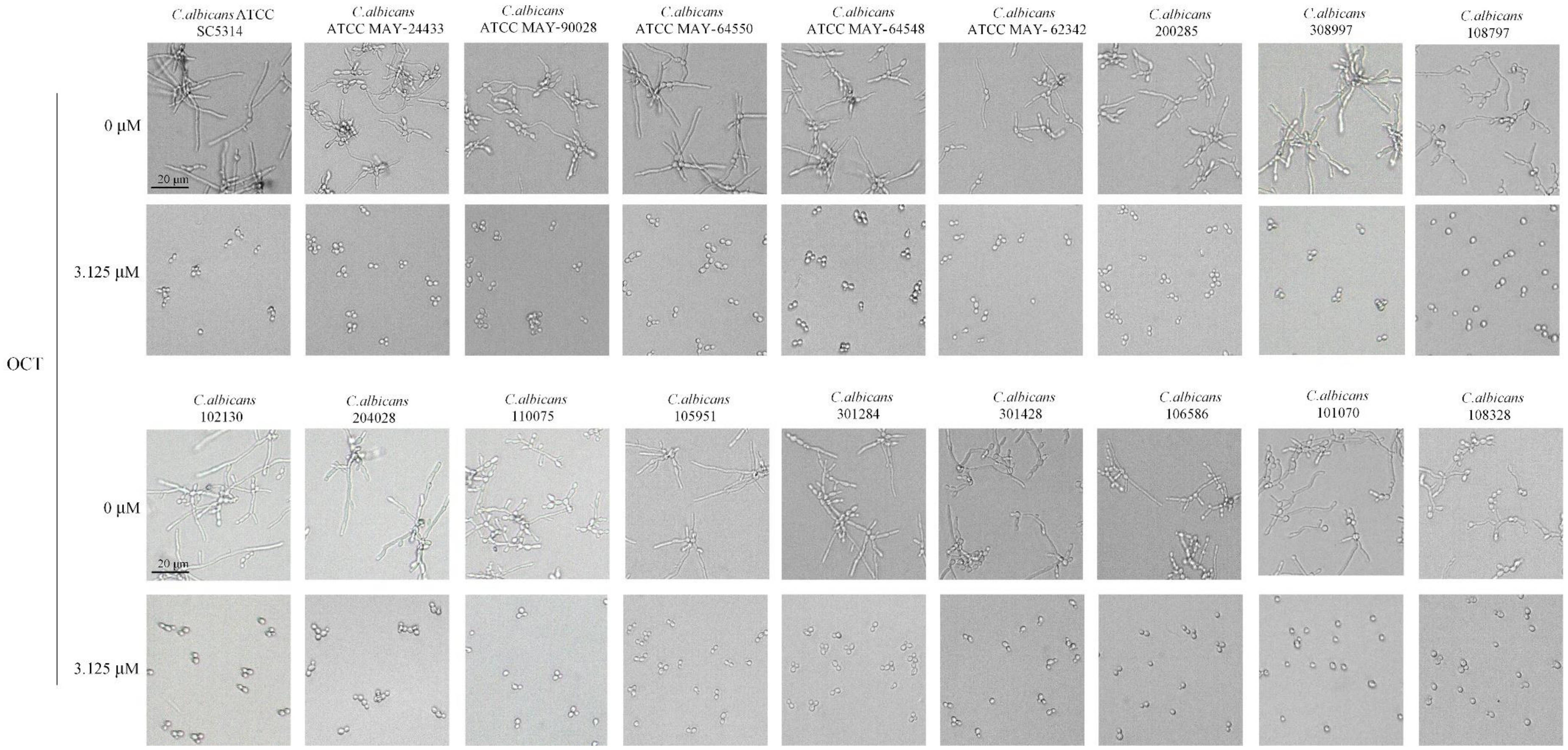

Figure S1. OCT inhibits filamentation of standard and clinical *C. albicans.* Cells were grown at 37°C for 4 h in the absence or presence of 3.125 μM OCT. Data represent two biological replicates.

Table S1. MHIC of 105 compounds

Product Name	MHIC (μM)	Target
Aclacinomycin A hydrochloride	25	Antibiotic; Proteasome; Topoisomerase
Almonertinib	50	EGFR
Almonertinib hydrochloride	50	EGFR
Asenapine hydrochloride	50	5-HT Receptor; Dopamine Receptor
Azaphen dihydrochloride monohydrate	100	Serotonin Transporter
Bazedoxifene acetate	100	Estrogen Receptor/ERR
Benfluorex hydrochloride	100	Others
Benzbromarone	100	Apoptosis; Xanthine Oxidase
Benzethonium chloride	12.5	nAChR
Bepridil hydrochloride hydrate	100	Calcium Channel
Bepridil hydrochloride	100	Calcium Channel
Bithionol	50	Parasite
Blonanserin	100	5-HT Receptor; Adrenergic Receptor; Dopamine Receptor; Sigma Receptor
Bronopol	100	Bacterial
Broxyquinoline	6.25	Parasite
Carmofur	50	Nucleoside Antimetabolite/Analog; SARS-CoV; Virus Protease
Carmustine	100	DNA Alkylator/Crosslinker
Celecoxib	100	COX
Ceritinib	25	ALK; IGF-1R; Insulin Receptor

Ceritinib dihydrochloride	25	ALK; IGF-1R; Insulin Receptor
Cetylpyridinium chloride monohydrate	12.5	Bacterial
Cetylpyridinium chloride	12.5	Bacterial; HBV
Chlorhexidine	25	Antibiotic; Bacterial
Chlorhexidine digluconate	100	Antibiotic; Bacterial
Chlormethine hydrochloride	100	Others
Chloroxine	6.25	Antibiotic; Bacterial
Chloroxylenol	100	Bacterial; Influenza Virus
Chlorprothixene	100	Bacterial; Dopamine Receptor; Histamine Receptor
Chlorquinaldol	25	Antibiotic; Bacterial; Fungal
Ciclopirox	25	Autophagy; Bacterial; Ferroptosis; Fungal
Ciclopirox olamine	25	Bacterial; Ferroptosis; Fungal
Cinacalcet	50	CaSR
Cinacalcet hydrochloride	50	CaSR
Clioquinol	6.25	Antibiotic; Autophagy; Fungal; Mitophagy
Clomiphene citrate	100	Estrogen Receptor/ERR
Crisaborole	100	Phosphodiesterase (PDE)
Cyclandelate	100	Others
Dacomitinib	100	Apoptosis; EGFR
Dalbavancin hydrochloride	50	Antibiotic; Bacterial
Demecarium bromide	100	AChE
Diclazuril	100	Antibiotic; Parasite

Diiodohydroxyquinoline	50	Antibiotic; Bacterial
Disulfiram	25	Aldehyde Dehydrogenase (ALDH); Interleukin Related; Pyroptosis
Domiphen bromide	12.5	Potassium Channel
Dronedarone	100	Adrenergic Receptor; Autophagy; Calcium Channel; Cytochrome P450; mAChR; Sodium Channel
Dronedarone hydrochloride	100	Autophagy; Potassium Channel
Efavirenz	100	Autophagy; HIV; Reverse Transcriptase
Efloxate	100	Others
Ethacridine lactate monohydrate	100	Bacterial
Fingolimod	50	LPL Receptor; PAK
Flupentixol dihydrochloride	50	Others
Hemin	100	Autophagy; Ferroptosis; Mitophagy
(+)-Kavain	100	Calcium Channel; GABA Receptor; Sodium Channel
Hexylresorcinol	100	Apoptosis; Bacterial; Glucosidase; Parasite
Lasofoxifene tartrate	100	Estrogen Receptor/ERR
Lomustine	50	Apoptosis; Autophagy; DNA Alkylator/Crosslinker
Magnolol	50	Autophagy; Bacterial; PPAR; RAR/RXR
Menadione	50	Endogenous Metabolite
Menadione bisulfite sodium	100	Others
Merbromin	50	Bacterial

Metergoline	100	5-HT Receptor; Dopamine Receptor; Sodium Channel
Miltefosine	50	Akt; HIV
Natamycin	25	Antibiotic; Endogenous Metabolite; Fungal
Netupitant	50	Neurokinin Receptor
Nitroxoline	25	Antibiotic; Autophagy; Bacterial
Octenidine dihydrochloride	3.125	Bacterial
Oritavancin diphosphate	100	Antibiotic; Bacterial
Osimertinib	50	EGFR
Osimertinib dimesylate	50	EGFR
Osimertinib mesylate	50	EGFR
Otilonium bromide	12.5	mAChR
Oxethazaine	100	HBV
Ozanimod	100	LPL Receptor
Perhexiline maleate	100	Others
Perphenazine	100	5-HT Receptor; Adrenergic Receptor; Dopamine Receptor; Histamine Receptor
Phenazine methylsulfate	100	Antibiotic; Apoptosis; Bacterial
Pimavanserin	100	5-HT Receptor
Pimavanserin tartrate	50	5-HT Receptor
Pinaverium bromide	100	Calcium Channel
Piroctone olamine	25	Fungal
Ponatinib	100	Autophagy; Bcr-Abl; FGFR; PDGFR; Src; VEGFR
Raloxifene hydrochloride	100	Autophagy; Estrogen Receptor/ERR

Riluzole	100	GABA Receptor; Sodium Channel
Rolapitant	100	Neurokinin Receptor
Salicylanilide	100	HIV; HIV Integrase
Sertindole	100	5-HT Receptor; Adrenergic Receptor; Autophagy; Dopamine Receptor
Sertraline hydrochloride	100	Serotonin Transporter
Silver sulfadiazine	12.5	Antibiotic; Bacterial; DNA/RNA Synthesis
Succimer	100	Others
Sultiame	100	Carbonic Anhydrase
Tafenoquine succinate	12.5	Parasite
Tamoxifen	50	Apoptosis; Autophagy; Estrogen Receptor/ERR; HSP
Tamoxifen citrate	50	Apoptosis; Autophagy; Estrogen Receptor/ERR; HSP
Tavaborole	3.125	Antibiotic; Fungal
Tegaserod maleate	100	5-HT Receptor
Thioridazine hydrochloride	100	5-HT Receptor; Autophagy; Dopamine Receptor
Thonzonium bromide	3.125	Bacterial; Proton Pump
Toremifene citrate	25	Apoptosis; Estrogen Receptor/ERR
Triclabendazole	100	Microtubule/Tubulin; Parasite
Triclosan	50	Antibiotic; Autophagy; Bacterial; Fungal
Triflupromazine hydrochloride	50	Autophagy; Dopamine Receptor
Visomitin	6.25	Reactive Oxygen Species
Vortioxetine	50	5-HT Receptor; Serotonin Transporter

Vortioxetine hydrobromide	50	5-HT Receptor; Serotonin Transporter
Zinc pyrithione	25	Bacterial; Fungal; Proton Pump

Table S2. Strains used in this study

No.	Strain	genotype	Source or reference
1	SN152	*his1 /his1 arg4/arg4 leu2/leu2*	Eukaryot Cell. 2005 Feb;4(2):298-309.
2	*hst7*Δ/*hst7*Δ	*hst7*::*HIS1* /*hst7*::*ARG4 leu2*/*leu2*	This study
3	*rim101*Δ/*rim101*Δ	*rim101*::*HIS1* /*rim101*::*ARG4 leu2* /*leu2*	
4	P_{ADH1}-*HST7*	*his1 /his1 ADE2 /ade2*:: P_{ADH1}-*HST7-ARG4 leu2/leu2*	
5	P_{ADH1}-*RIM101*	*his1 /his1 ADE2 /ade2*:: PADH1-*RIM101-ARG4 leu2/leu2*	
6	*erg1*Δ/*erg1*Δ	*erg1*::*HIS1* /*erg1*::*ARG4 leu2* /*leu2*	mBio. 2023 Jan-Feb; 14(1): e02639-22.
7	*erg2*Δ/*erg2*Δ	*erg2*::*HIS1* /*erg2*::*ARG4 leu2* /*leu2*	
8	*erg3*Δ/*erg3*Δ	*erg3*::*HIS1* /*erg3*::*ARG4 leu2* /*leu2*	
9	*erg4*Δ/*erg4*Δ	*erg4*::*HIS1* /*erg4*::*ARG4 leu2* /*leu2*	
10	*erg5*Δ/*erg5*Δ	*erg5*::*HIS1* /*erg5*::*ARG4 leu2* /*leu2*	
11	*erg6*Δ/*erg6*Δ	*erg6*::*HIS1* /*erg6*::*ARG4 leu2* /*leu2*	
12	*erg24*Δ/*erg24*Δ	*erg24*::*HIS1* /*erg24*::*ARG4 leu2* /*leu2*	
13	*erg25*Δ/*erg25*Δ	*erg25*::*HIS1* /*erg25*::*ARG4 leu2* /*leu2*	
14	*upc2*Δ/*upc2*Δ	*upc2*::*HIS1* /*upc2*::*ARG4 leu2* /*leu2*	

Table S3. Primers used in this study

No.	Primer name	Primer sequence (5'to3')
		**Primers for genes deletion**
1	Hst7P1	ATAGAACTCCCACAGGCGCT
2	Hst7P3	cacggcgcgcctagcagcggTGTTGAGGTGGAGTATGAAT
3	Hst7P4	gtcagcggccgcatccctgcCACAACTCATATACATATAC
4	Hst7P6	CAAGAGATGTTCAACTACAT
5	Hst7Upcheck	TATAAGTTGCGACGAAGCCT
6	Hst7Dncheck	TTTTGTTGGATTTGGCTGAT
7	Rim101P1	CTTGGCAATCAATTCCCAGA
8	Rim101P3	cacggcgcgcctagcagcggTGTCTAAAAATCTCGTTTGT
9	Rim101P4	gtcagcggccgcatccctgcGCAATTATACCGATTTTACT
10	Rim101P6	GTTGTGTCTCCAATCATTGC
11	Rim101Upcheck	TGTACTCACTCACAGCCCCT
12	Rim101Dncheck	AGGTCACGATAACCCCAAGT
13	ARG4 Left	TTCAACCTTTCAAACGATGC
14	ARG4 Right	TCGATACATTTGCGGTACAG
15	HIS1 Left	ATTAGATACGTTGGTGGTTC
16	HIS1 Right	AACACAACTGCACAATCTGG
17	Universal 2	ccgctgctaggcgcgccgtgACCAGTGTGATGGATATCTGC
18	Universal 5	gcagggatgcggccgctgacAGCTCGGATCCACTAGTAACG
		**Primers for genes overexpression**
19	Rim101F1	ATAGATATTACATACAAGTTTTTAACCTAGACAAACGAGTGCCACCTGACGTCTAAGAA
20	Rim101F2	TCTTTCAACAACTAACTAATTTTATACTATCCACCAACTATAGATATTACATACAAGTT
21	Rim101R1	AGCATTTAAGTATGTTACGGGATGAATGTTGTAATTCATCGGTATGACCATGATTACCT

22	Rim101R2	GTGACTTGCAGTACTCTCACTTGCACCGGTATTGCTATCAGCATTTAAGTATGTTACGG
23	Tet-Rim101Dncheck	TGGGTATAAGATTGCAGAGC
24	Hst7F1	TTGTTCATTGTTTTTGCTCATTCATACTCCACCTCAACATGCCACCTGACGTCTAAGAA
25	Hst7F2	TTGAATATATTAATAATAAAATTATAATAACAGGTTTGCTTGTTCATTGTTTTTGCTCA
26	Hst7R1	TGTAGCTTCTTGTGTATCTATACGAGTTGTTCTTGTCATCGGTATGACCATGATTACCT
27	Hst7R2	TGATAATGGTGAAGGCACTGGTGGTAAATCCTTATGCTTTGTAGCTTCTTGTGTATCTA
28	Tet-Hst7Dncheck	AGGGGATTTCTGTGCAAATT
29	VP16	AATGCCAATACTCCTCTTTCTC
30	VP17	TCAACAAACTCTTTCAACTTCTCA
